# The role of the peripheral system dysfunction in the pathogenesis of sepsis-associated encephalopathy

**DOI:** 10.3389/fmicb.2024.1337994

**Published:** 2024-01-17

**Authors:** Jingyu Zhang, Shuangli Chen, Xiyou Hu, Lihong Huang, PeiYong Loh, Xinru Yuan, Zhen Liu, Jinyu Lian, Lianqi Geng, Zelin Chen, Yi Guo, Bo Chen

**Affiliations:** ^1^Research Center of Experimental Acupuncture Science, Tianjin University of Traditional Chinese Medicine, Tianjin, China; ^2^School of International Education, Tianjin University of Traditional Chinese Medicine, Tianjin, China; ^3^Binhai New Area Hospital of TCM, Fourth Teaching Hospital of Tianjin University of TCM, Tianjin, China; ^4^Tianjin Key Laboratory of Modern Chinese Medicine Theory of Innovation and Application, Tianjin University of Traditional Chinese Medicine, Tianjin, China; ^5^School of Acupuncture and Moxibustion and Tuina, Tianjin University of Traditional Chinese Medicine, Tianjin, China; ^6^National Clinical Research Center for Chinese Medicine Acupuncture and Moxibustion, Tianjin, China

**Keywords:** sepsis-associated encephalopathy, glial cells, intestinal flora, peripheral immune cells, exosomes

## Abstract

Sepsis is a condition that greatly impacts the brain, leading to neurological dysfunction and heightened mortality rates, making it one of the primary organs affected. Injury to the central nervous system can be attributed to dysfunction of various organs throughout the entire body and imbalances within the peripheral immune system. Furthermore, central nervous system injury can create a vicious circle with infection-induced peripheral immune disorders. We collate the pathogenesis of septic encephalopathy, which involves microglial activation, programmed cell death, mitochondrial dysfunction, endoplasmic reticulum stress, neurotransmitter imbalance, and blood–brain barrier disruption. We also spotlight the effects of intestinal flora and its metabolites, enterocyte-derived exosomes, cholinergic anti-inflammatory pathway, peripheral T cells and their cytokines on septic encephalopathy.

## 1 Introduction

The brain is often considered the first organ to be exposed to inflammation. Sepsis, a condition characterized by an uncontrolled immune response to infection, is defined as life-threatening organ dysfunction. Within this context, the occurrence of brain injury emerges as a frequently observed complication (Singer et al., 2016). Sepsis-associated encephalopathy (SAE) refers to a condition characterized by widespread cognitive impairment resulting from the systemic inflammatory reaction triggered by diverse infections. The main manifestation is an altered state of consciousness (Molnár et al., 2018). High prevalence of SAE is observed, reaching 70%, in patients suffering from severe systemic infection while admitted to the intensive care unit (Gofton and Young, 2012). SAE is closely associated with increased mortality, high hospital costs and prolonged hospital stays, persistent cognitive impairment and limited physical function (Ren et al., 2020). The underlying mechanism of SAE remains undetermined. Current studies suggest that SAE may be caused by inflammation activated by endothelial/ microglia, increased blood–brain barrier permeability, hypoxia, neurotransmitter imbalance, loss of axons and neurons. The clinical treatment of SAE mainly centers on the rapid optimization of antibiotics, such as statins, levodopa/benserazide, and bactericidal non-dissolving antibiotics. Animal experiments focus on regulating inflammation, stabilizing of the blood–brain barrier and rehabilitating mitochondrial function (Tauber et al., 2021).

The central nervous system (CNS) is widely recognized as an essential component in maintaining the proper functioning of the immune system. It can manipulate systemic inflammatory signals and immune regulation through multiple pathways, including cholinergic anti-inflammatory pathway (Olofsson et al., 2012). SAE-induced brain dysfunction can lead to abnormal responses of multiple neuroendocrine immune networks. Brain injury is a crucial factor in the prognosis and survival of sepsis patients and should be acknowledged not only as an affected organ but also as a significant contributor to the compromised immune regulation resulting from sepsis (Lee et al., 2010). In SAE research, two main models are commonly used. The first model involves cecal ligation and puncture (CLP), while the second model involves intraperitoneal injection of a specific dose of lipopolysaccharide (LPS). The brain regions that have received the most attention in these models are the cerebral cortex and hippocampus. Therefore, we have summarized the changes observed in these brain regions, as well as in the intestines, liver, and peripheral immune system, in the SAE model. Furthermore, relevant clinical studies are also discussed. In short, this paper summarizes the existing studies on the pathogenesis of sepsis on the one hand, and explores the interactions between the brain, intestine, liver and peripheral immune system on the other hand. Furthermore, it can provide theoretical basis for the clinical treatment of SAE.

## 2 Pathogenesis of sepsis-associated encephalopathy

### 2.1 Neuroinflammation

Sepsis-induced central nervous system (CNS) neuroinflammation has been considered as an underlying mechanism of delayed cognitive impairment (Xing et al., 2018). Systemic inflammation during sepsis spreads to the brain through damaged blood–brain barrier (BBB) (Obermeier et al., 2013), thereby activating resting glial cells. Activated glial cells can be found even under circumstances where there is no obvious BBB damage (Griton et al., 2020). These cells release inflammatory factors, which aggravate neuroinflammation, thus forming a vicious circle (Faraco et al., 2007). Microglia, which are crucial components of the innate immune system in the brain, serve as significant contributors to the production of pro-inflammatory cytokines. Consequently, they gain primary attention in the investigation of neuroinflammation. Microglia can be swiftly triggered by diverse stimuli, encompassing infectious or pathological stimuli, along with Aβ peptides. Once activated, microglia have the capability to release substantial quantities of pro-inflammatory cytokines, such as TNF-α, IL-6, and IL-1β. This augmented neuroinflammation within the brain exacerbates neuronal harm, leading to the manifestation of SAE’s behavioral and psychological symptoms (Ye et al., 2019). A prospective autopsy study was conducted to compare microglia numbers in specific brain regions between 16 well-characterized patients with septic shock and 15 controls. The study revealed that patients who died during septic shock and had systemic inflammation exhibited higher numbers of CD-68 microglia in the putamen, cerebellum, and hippocampus. This finding suggests that severe systemic inflammation triggers a neuroinflammatory response characterized by the activation of microglia (Westhoff et al., 2019).

The receptors on the surface of neuroglial cell have been found to be involved in the occurrence of neuroinflammation. The study found that both astrocytes and microglia (increased Iba-1 and C3) were activated in SAE. Intracerebroventricular administration of BRL-44408, a specific antagonist of α2A adrenergic receptors, to CLP mice led to the upregulation of pro-inflammatory cytokines, including TNF-α, IL-6, and IL-1β, in the hippocampus. Neuroinflammation was effectively suppressed by dexmedetomidine, a remarkably selective α2-adrenoceptor agonist. However, the therapeutic effect of dexmedetomidine remained intact even after depleting microglia. This indicates the crucial involvement of astrocytes, particularly in the expression of α2A-adrenoceptors, rather than microglia in neuroinflammation (Mei et al., 2021). In SAE, the NLRP3 inflammasome in microglia is activated and promotes cleavage of procaspase-1. Mature caspase-1 cleaves pro-IL-1β to IL-1β, thereby promoting neuroinflammation (Swanson et al., 2019). A neuroprotective molecule, indole-3-propionic acid (IPA), inhibited the levels of NLRP3 and IL-1β in the cortex. Vitro experiments found that IPA can inhibit the increase in levels of NLRP3 and IL-1β in primary microglial cells stimulated by LPS. The therapeutic effect of IPA was weakened by an aryl hydrocarbon receptor (AhR) inhibitor (CH223191). This suggests that AhR on microglia may play a crucial role in neuroinflammation (Fang et al., 2022). Furthermore, microglia are the major resident immune cells in the CNS. These unique cells possess the remarkable capability to undergo morphological and functional adjustments in response to alterations in their surrounding microenvironment. In instances of sepsis, a transition from a state of “surveillance” to a pro-inflammatory M1 phenotype occurs. This change prompts the release of inflammatory signals, as evidenced by observations in the hippocampus of CLP mice and in primary microglia cultures stimulated with LPS (Shen et al., 2021). Moreover, in addition to glial cells, many immune cells play regulatory roles in infection-induced neuroinflammation. The brain of mice displayed the infiltration of neutrophils and T cells following sepsis for 10 days. This infiltration was accompanied by an augmentation of regulatory T cells (Treg) and Th2 cells, which played a crucial role in the mitigation of SAE (Saito et al., 2021).

The interplay between oxidative stress and inflammatory responses is evident. The inflammatory response triggers oxidative stress, which in turn intensifies the inflammatory response (Liu et al., 2023). Notably, oxidative stress plays a pivotal role in the pathophysiology of SAE. Analysis of brain tissue sections of CLP mice using fluorescent double staining exhibits the co-localization of the microglia marker protein IBA-1 with the protein inducible nitric oxide synthase (i-NOS) and Cyclooxygenase-2 (COX-2). The Nrf2/HO-1 pathway is a crucial mechanism for combating oxidative stress. When there is oxidative stress, Nrf2 is activated to regulate the gene expression of antioxidant proteins in cells. HO-1, a downstream target protein of Nrf2, also functions as an antioxidant (Ali et al., 2018; Korytina et al., 2019). The Western blot analysis revealed a moderate upregulation of Nrf2 and HO-1 expression in primary microglia treated with LPS. However, the levels of Nrf2 and HO-1 were significantly increased upon administration of sodium butyrate. These findings suggest that the Nrf2/HO-1 pathway plays a crucial role in regulating oxidative stress in microglia (Zhang H. et al., 2022). Nrf2 also inhibits the activation of the NLRP3 pathway in glial cells, thus leading to a reduction in neuroinflammation (Xie K. et al., 2020). In another animal experiment, little damage was observed to the BBB in rats with CLP after 24 h, suggesting that peripheral inflammatory cytokines such as IL-1β may not be able to enter the CNS at an early stage. The expression of IL-1β was upregulated in microglia and cerebral microvascular endothelial cells (CMECs), as detected by Western blot, ELISA and immunofluorescence co-localization techniques. The use of Fisetin to reduce IL-1 expression in CMECs limits its binding to IL-1R1 in microglia. This supports that IL-1β in microglia may originate from CMECs. IL-1R1/pNF-κB pathway further activates microglia, leading to neuroinflammation and cognitive impairment (Ding et al., 2022). Foxc1 is a transcription factor that can suppress oxidative stress and inflammation (Xia et al., 2019). In a study, it was discovered that the levels of Foxc1 and IκBα were reduced in the hippocampus of CLP mice. However, in mice with excessive Foxc1 expression, the overexpression of Foxc1 was found to hinder the migration of microglial cells, inflammation, and neuronal apoptosis in the hippocampus of CLP mice. This effect was achieved through the IκBα/NF-κB pathway. In addition, the expressions of Foxc1 and NF-κB inhibitor (IκBα) were significantly decreased, while the expressions of p65, IL-1β, and TNF-α were markedly elevated in microglia treated with LPS. After overexpressing Foxc1 through adenovirus transfection, the expression of IκBα was upregulated, while that of p65, IL-1β and TNF-α was downregulated. In addition, knockdown of IκBα resulted in a significant increase in IL-1β and TNF-α levels, as well as promotion of microglial migration. It was indicated that the diminished presence of Foxc1 within LPS-exposed microglia fosters neuroinflammation through the suppression of IκBα and the initiation of the NF-κB pathway (Wang et al., 2022b).

MicroRNAs (miRNAs) are a widely distributed group of small, non-coding RNA molecules that are single-stranded and have a crucial role in various pathological conditions (Fabbri et al., 2012). The study findings demonstrated a significant increase in RNA and miRNA levels in the plasma of CLP mice for seven consecutive days. This increase specifically included miR-146a, miR-145, miR-34a, and miR-122. *In vitro* experiments conducted on cultured microglia and astrocytes demonstrated a substantial and proportional reaction of IL-6 and CXCL2 upon exposure to synthetic miR-146a, miR-145, miR-34a, or miR-122. Furthermore, the absence of the TLR7 gene displayed a significant reduction in the expression of cytokines and activation of microglia. It was also observed that TLR7 was more prevalent in microglia in comparison to astrocytes. These studies propose that the activation of the miR-146a-5p stimulates the innate immune responses in the brain through the TLR7 pathway in microglia (Zou et al., 2022).

Autophagy is a process of catabolism that is evolutionarily conserved, aiming to recycle proteins and organelles that are damaged or become senescent. By increasing autophagy, there is a compensatory response that intends to restrict sepsis-driven harm to tissues (Tanida et al., 2008). Inhibiting mTOR can promote autophagy, which affects immune response and cytokine secretion processes (Weichhart, 2018). The study observed an increase in TNF-α, IL-6, HMGB1, and M1 type microglia in the hippocampus of CLP mice. The ratio of p-mTOR/mTOR and the expression of p62 were upregulated in LPS-treated microglia (BV-2 cells). Hydrogen-rich medium can regulate microglial polarization and reduce neuroinflammation through mTOR inhibition and autophagy (Zhuang et al., 2020). The findings from an another investigation in CLP mice models and primary microglia cultures indicated that sepsis elevated the expression of hippocampal CXCR5, leading to incomplete initiation of autophagy, polarization of microglia towards the M1 phenotype, generation of inflammatory cytokines, and manifestation of cognitive impairments. The downregulation of CXCR5 serves to reinstate autophagy, shift microglia towards an M2 phenotype, and suppress p38MAPK/NF-κB/STAT3 signaling, ultimately mitigating sepsis-induced neuroinflammation and cognitive deficits (Shen et al., 2021). Limited knowledge exists regarding the involvement of autophagy in the degradation of nuclear components in SAE. A study has identified the co-localization of the autophagy marker LC3B with the nuclear marker laminin B1 in the hippocampus of septic mice, suggesting the presence of nuclear autophagy (Xie et al., 2022).

### 2.2 Neuronal damage

Pyroptosis, which is also recognized as cellular inflammatory necrosis, manifests through the ongoing enlargement of cells until the point when the cell membrane ruptures, leading to the discharge of cellular components and initiation of an intense inflammatory reaction (Jorgensen and Miao, 2015). An overabundance of pyroptosis can pose a threat to the overall well-being of tissues and cells. In order to safeguard the host organism against harm, the regulation of pyroptosis is carried out with precision, with the involvement of inflammatory caspases like caspase-1, caspase-4, caspase-5, and caspase-11. It is generally considered to be induced by the cleavage of GSDMD by caspase-1 and other caspases. The study found that the downregulation of caspase-1 can inhibit GSDMD and its cleaved form GSDMD-NT expression, reduce brain pyroptosis and protect synaptic plasticity (Xu et al., 2019). Moreover, the investigation revealed a notable escalation in the quantity of NLRP3 and caspase-1 positive cells within the CA1 area of the murine cerebral cortex following a span of 7 days of CLP, as illustrated by means of immunohistochemical analyses. Conversely, Inhibition of NLRP3 (using MCC950) or caspase-1 (using Ac-YVAD-CMK) modulates pyroptosis by regulating GSDMD expression, and inflammatory responses by regulating IL-1β and IL-18 expression, in the hippocampus of CLP mice, thereby alleviating its impact. These findings solidify the proposition that sepsis can prompt the activation of the NLRP3 inflammasome pathway, consequently leading to the activation of caspase-1 and instigation of inflammatory cascade reactions and pyroptosis (Fu et al., 2019). NLRP3 has the capability to trigger apoptosis and pyroptosis via an intricate molecular mechanism. Maf1 serves as a conserved inhibitor of RNA polymerase (pol) III (RNAP III). Studies have shown that overexpression of Maf1 directly binds to the NLRP3 promoter, resulting in inhibition of NLRP3 inflammasome formation and pro-inflammatory protein release. Furthermore, Maf1 competitively inhibits the binding of NF-κB/p65 to the NLRP3 promoter. This inhibits the expression of inflammation-activated pyroprotein (GSDMD), upregulates the levels of antiapoptotic protein Bcl-2, and downregulates proapoptotic protein Bax, thereby ameliorating NLRP3 inflammasome-induced apoptosis and pyrolysis (Chen S. et al., 2020).

Ferroptosis is a type of cell death and instead relies on the buildup of iron within cells, resulting in higher levels of harmful lipid peroxide ROS. Studies have found that SAE triggers hippocampal ferroptosis, which involves an increase in ROS, iron content, as well as malondialdehyde (MDA). Additionally, there is a decrease in glutathione (GSH) levels, and changes in the expression of ferroptosis-associated proteins (GPX4, ACSL4, and SLC7A11). Ferroptosis in hippocampal cells also triggers the recruitment of microglia, promoting an inflammatory microenvironment in SAE (Wang J. et al., 2022). Exosomes, which range in size from 30 to 150 nm, are small vesicles composed of a lipid membrane that carry a diverse array of complex molecules, including proteins and various forms of RNA, both coding and non-coding. NEAT1 binds to hsa-miR-9-5p, which targets the genes for transferrin receptor (TFRC) and glutamate-oxaloacetate transaminase 1 (GOT1). The transferrin receptor TFRC is responsible for transporting iron ions from outside the cell (Gammella et al., 2017; Xie et al., 2019). Recent research has revealed that sepsis can stimulate a substantial increase in exosome-associated lncRNA NEAT1. Moreover, these exosomes play a crucial role in transporting NEAT1 across the blood–brain barrier (BBB) and into the cerebral cortex. The implication of this mechanism is that it contributes to the exacerbation of severe acute encephalopathy (SAE) by inducing ferroptosis, a specific type of cell death, in brain microvascular endothelial cells. This damaging effect is achieved through the regulation of miR-9-5p, TFRC, and GOT1 axis (Wei et al., 2022).

During the initial stages of neural development in mice, the involvement of SOX2OT in the transcriptional regulation of embryonic neurogenesis processes can be observed (Tosetti et al., 2017). Research investigations conducted demonstrated that mice displayed a gradual elevation in the levels of both SOX2OT and SOX2 mRNA at distinct time points following CLP surgery, namely days 3, 7, and 14. Furthermore, this increase in expression was found to be associated with a decline in cognitive function. Analysis using immunofluorescence techniques indicated a reduction in neuronal markers (BrdU+/DCX+, BrdU+/NeuN+) within the dentate gyrus of the hippocampus, implying a decrease in the overall number of neurons. However, when the expression of SOX2OT was suppressed, it was observed that cell proliferation and survival in mature neurons were restored, resulting in an improvement in cognitive impairment (Yin et al., 2020). Tau protein has been investigated as a biomarker for brain injury. In a retrospective observational study, it was found that the average serum tau protein level in the group with SAE was significantly higher than in the non-SAE group. There was a strong correlation between serum tau protein levels in patients with severe sepsis and the occurrence of SAE (Zhao et al., 2019). In animal experiments, it was observed that the levels of ptau and ptau key kinases were elevated in the hippocampus of CLP mice. Studies have also found decreased dendritic spine density and the number of normal hippocampal neurons in CLP mice, indicating damage to neuronal synapses (Qi et al., 2023). Glutamate serves as the foremost excitatory neurotransmitter within the central nervous system, while the critical function of ionotropic glutamate receptors (NMDAR) involves the regulation of neuronal survival and synaptic plasticity. The abundance of synaptic glutamate can excessively activate NMDAR, thus resulting in excitotoxicity and subsequent harm to nerve cells (Asada et al., 2022). The research has shown that CLP rats exhibit elevated levels of glutamate in the hippocampus, in conjunction with an upregulated expression of the NMDAR1 glutamate receptor (Tang et al., 2023). Syntaxin1A primarily serves as an indicator for synaptic vesicles abundance in synaptically active regions (Ullrich et al., 2015). Munc18-1 is a crucial protein that is encoded by the STXBP1 gene. It plays a significant role in synaptic vesicle docking and fusion by interacting with Syntaxin1A. As a result, it has a direct impact on neurotransmitter transmission (Romaniello et al., 2015). Synapsin also plays a crucial function in synaptic vesicle trafficking, docking, and fusion, which holds significant significance (Evans and Cousin, 2005). The studies have found that the expressions of Munc18-1, Syntaxin1A and synapsin increased in the hippocampus of septic rats. Additionally, the expression levels of Syntaxin1A, synapsin and glutamate decreased after interference with Munc18-1 siRNA. These indicated that Munc18-1 may affect glutamate levels by regulating Syntaxin1A and synapsin, thereby participating in the process of hippocampal injury in septic rats (Tang et al., 2023).

### 2.3 Organelle dysfunction

A Retrospective cohort study found that Variations in mtDNA are associated with development of and protection from delirium during sepsis. This study highlights the role of mitochondrial dysfunction in sepsis as a crucial factor contributing to sepsis-related delirium (Samuels et al., 2019). A clinical study conducted on peripheral blood samples collected from 20 premature infants revealed a potential correlation between high expression of miRNA-1197 and low expression of miRNA-485-5p with the pathogenesis of oxidative respiratory chain and energy metabolism in premature infants with SAE. These findings suggest a possible association with mitochondrial dysfunction during SAE (Gong et al., 2022). Common neuroinflammation often affects mitochondrial health in SAE. Animal Studies have shown that the upregulation of NLRP3 and Nrf2 in microglia leads to mitochondrial dysfunction. Hydrogen can alleviate and improve mitochondrial function by inhibiting the Nrf2-mediated NLRP3 pathway (Xie K. et al., 2020). In experiments conducted on cell cultures, it has been observed that exposure to lipopolysaccharide (LPS) leads to a decrease in mitochondrial membrane potential (MMP) in hippocampal neuron cell lines (HT-22), which suggests the presence of mitochondrial dysfunction (Wang J. et al., 2022). Mitochondrial dysfunction plays a crucial role in sepsis-induced multiple organ failure, resulting in cytopathic hypoxia that impairs normal cellular function (Singer, 2014). After that, mitochondrial damages occur. The transmission electron microscope showed that the damaged mitochondria in the brain tissue were swollen and vacuolated. The damaged mitochondria also caused the accumulation of ROS, which further activated NLRP3 and aggravated the inflammatory response. Hence, alterations in the dynamics of mitochondria emerge as imperative for regulating the quality control of mitochondria amidst sepsis. Research has discovered that primary neurons treated with LPS undergo a transition from oxidative phosphorylation to glycolysis. This results in a reduction of both ATP production and MMP. Additionally, the expression of dynein-related protein 1 (Drp1) undergoes an elevation. While an inhibitor of the Drp1-Fis1 (P110) effectively improved mitochondrial health, suggesting that Drp1-Fis1 mediates mitochondrial dysfunction in SAE (Haileselassie et al., 2020). Mitophagy acts as a crucial autophagic mechanism in preserving cellular homeostasis and disposing of impaired mitochondria (Doblado et al., 2021). However, mitophagy impairment may occur in SAE. Research has observed that the utilization of fisetin-induced medications has the ability to trigger mitophagy, which facilitates the elimination of impaired mitochondria and ROS during sepsis. Specifically, Fisetin enhances mitophagy in CMECs of CLP rats by increasing the expression of LC3-II, decreasing the levels of p62, and reducing ROS (Ding et al., 2022).

Excessive activation of endoplasmic reticulum (ER) stress is significantly associated with the cellular harm triggered by sepsis. ER stress occurs due to physiological or pathological events that disturb normal protein folding within the ER, giving rise to the unfolded protein response (UPR) (Sun et al., 2020). However, excessive UPR responses beyond cellular adaptation can lead to apoptosis (Lin et al., 2008). Therefore, maintaining normal levels of ER stress plays a crucial role in reducing sepsis-induced tissue damage (Gupta et al., 2010). Studies have found that the expression levels of UPR markers (GRP78, CHOP and PERK) significantly increased in two neuronal cell lines (PC12 and MES23.5) treated with LPS, decreased cell viability, and enhanced apoptosis. Furthermore, the expression levels of GRP78 increased with increasing doses of LPS treatment. Moreover, the overexpression of GRP78 led to decreased cell viability and increased apoptosis over time in both cell lines. These findings suggest that LPS-induced ER stress can promote apoptosis as a cellular adaptive response (Li et al., 2020).

### 2.4 Imbalance of neurotransmitters

Neurotransmitters are a group of chemical substances that transmit information between presynaptic and postsynaptic neurons. They can be divided into two types: excitatory neurotransmitters and inhibitory neurotransmitters. The imbalance of neurotransmitter may be associated with sepsis-associated encephalopathy (Tang et al., 2022). The main manifestations include increased levels of glutamate, decreased levels of acetylcholine, and reduced levels of γ-aminobutyric acid, etc. Restoring the balance of neurotransmitters could potentially be a target for treating SAE.

Glutamate, the primary excitatory neurotransmitter in the brain, can become excessively accumulated and result in neuronal excitotoxicity. A research study utilized a specialized imaging tool called glutamate-weighted chemical exchange saturation transfer (GluCEST) to identify alterations in glutamate signaling caused by neuroinflammation. The study revealed a substantial rise in glutamate levels in the hippocampus of rats with LPS-induced sepsis, which can potentially lead to disruptions in the neurotransmitter system. This observation sheds light on the detrimental effects of glutamate accumulation in the context of neuroinflammatory conditions (Lee et al., 2023). Another study found that inhibiting ferroptosis can alleviate cognitive disorders and neurological impairments in mice with sepsis-associated encephalopathy. Additionally, inhibiting ferroptosis can attenuate glutamate excitotoxicity induced by ferroptosis, thereby protecting the integrity of synapses and neurons (Xie et al., 2022). Its mechanism may also be related to the cystine/glutamate antiporter (System Xc-), which is a Na^+^-independent reverse transport protein across the cell membrane. Its function is to uptake cystine and excrete glutamate. Redox imbalance occurs in cells with ferroptosis, which leads to the upregulation of System Xc- and excess accumulation of glutamate in the synaptic cleft, triggering excitotoxicity (Sato et al., 1999; Ogunrinu and Sontheimer, 2010). Furthermore, the prefrontal cortex (PFC)-hippocampus (HPC) pathway holds significant significance in cognitive functions, encompassing attention, decision-making, and both immediate and enduring memory (Jones and Wilson, 2005). One study found that cognitive dysfunction in CLP-induced septic mice was enhanced by chemical genetic activation of the HPC-PFC pathway, which could be blocked by glutamate receptor antagonists (Ge et al., 2023).

Acetylcholine is also an excitatory neurotransmitter, which differs from the excessive accumulation of glutamate. SAE exhibit a decrease in the expression of acetylcholine receptors within the hippocampus, leading to the inhibition of the cholinergic anti-inflammatory pathway in the vagus nerve. Consequently, this disruption triggers an unregulated inflammatory reaction and impairs neurological functionality (Hong et al., 2023). A study has found that defects in cholinergic nerve function and abnormal neuroinflammation have a synergistic effect on the pathogenesis of SAE. SAE rats exhibit defects in cholinergic neurological function, accompanied by overexpression of pro-inflammatory cytokines, increased neuronal apoptosis, and cognitive impairment in the brain. The acetylcholinesterase inhibitor huperzine (HupA) significantly improved cholinergic nerve function, attenuated abnormal neuroinflammation in SAE, and restored brain function (Zhu et al., 2016). In another investigation, murine p75-saporin immunotoxin (mu-p75-sap) was employed to induce specific harm to the cholinergic system within the basal forebrain of mice. It was discovered that animals with cholinergic defects exhibited acute and transient impairments in working memory when exposed to low-dose LPS, while control groups with impaired showed no effect. It has indicated that experiencing cholinergic depletion increases susceptibility to acute cognitive impairments that may arise after subsequent systemic inflammatory injuries (Field et al., 2012).

γ-Aminobutyric acid (GABA) is an inhibitory neurotransmitter. Parvalbumin (PV) interneurons are the most dominant subtype among GABAergic interneurons (Enwright et al., 2016). Most cortical PV interneurons are wrapped by a perineuronal net (PNN). PNN is a condensed form of extracellular matrix (Berretta et al., 2015) that is involved in the closure of developmental critical periods, regulates synaptic plasticity, and can be altered by oxidative stress. PNN plays a role in protecting and regulating PV interneurons (McRae and Porter, 2012; Cabungcal et al., 2013). One study found that mice treated by LPS exhibited significant cognitive disorder, which was associated with reduced densities of PNN and PV neuron. Active MMP-9-mediated PNN remodeling results in a decrease in inhibitory and excitatory inputs to PNN-wrapped PV interneurons, as well as a reduction in gamma oscillations in the hippocampal CA1 region (Zhang et al., 2023). This suggests that sepsis leads to decreased release of the inhibitory neurotransmitter GABA.

### 2.5 Blood–brain barrier disruption

**Figure 1:**
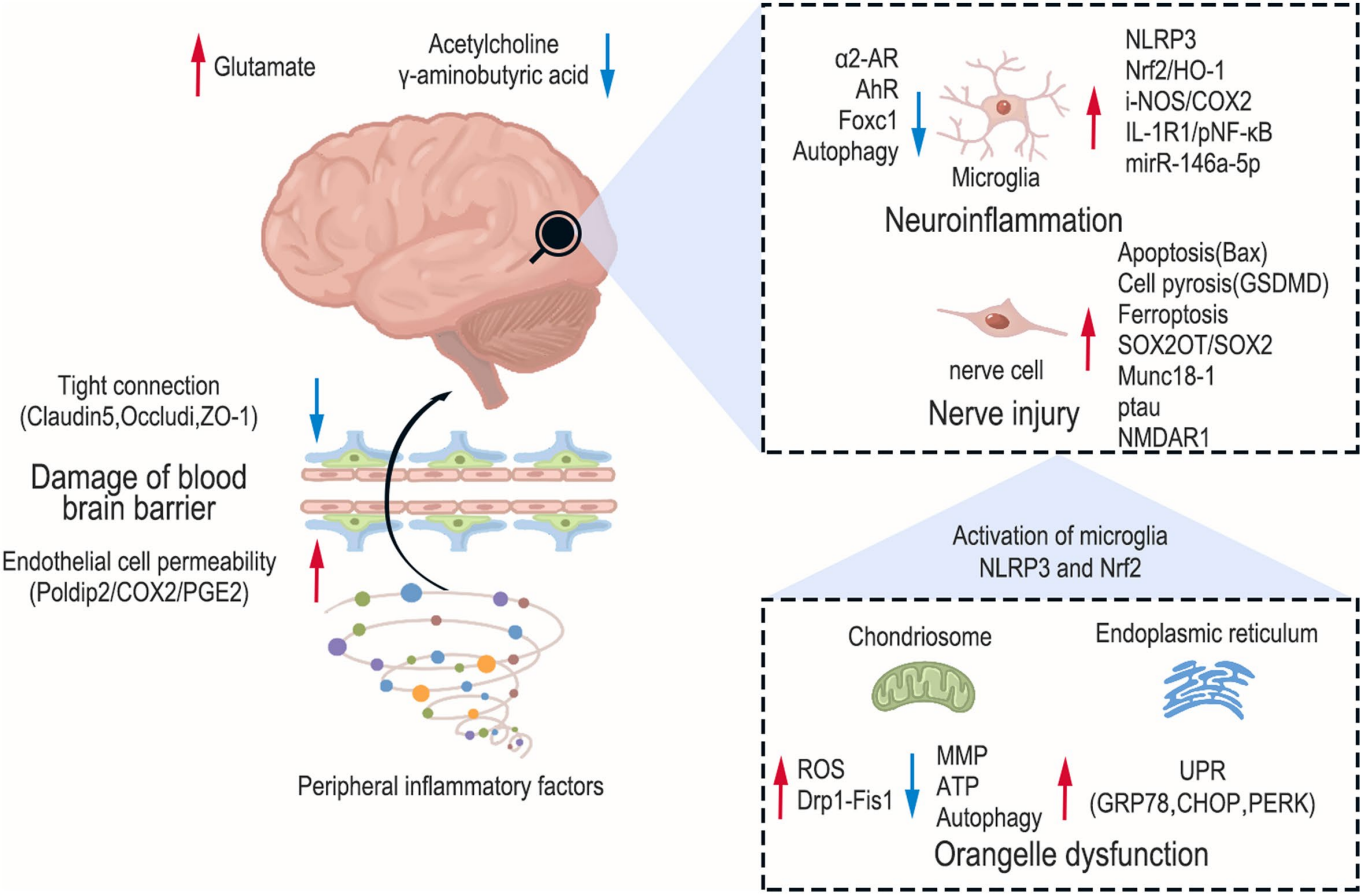
Pathogenesis of sepsis-associated encephalopathy. The red arrows indicate upward regulation and blue arrows indicate downward regulation. The brain sustains damage at various levels during SAE, encompassing brain neurotransmitter imbalance, activation of glial cells, mitochondrial dysfunction, and endoplasmic reticulum stress. Furthermore, peripheral inflammation can also compromise the permeability of the blood–brain barrier.

The BBB, a dynamic “physical barricade,” manages the movement of molecules between the brain and blood, thus preserving CNS stability (Sweeney et al., 2019). The components of the BBB encompass endothelial cells, astrocyte endfeet, and tight junctions (Abbott et al., 2010). Inflammation is the primary factor in BBB disruption in SAE. We have previously highlighted glial cells in neuroinflammation. Astrocyte activation and brain-derived inflammatory factors appear to be among the causes of BBB disruption. Inflammation can also contribute to BBB disruption in sepsis by altering endothelial cell permeability. It has been reported that physiological doses of prostaglandin E2 (PGE2) are sufficient to induce brain endothelial cell permeability *in vitro* (Dalvi et al., 2015). After LPS treatment, the multifunctional protein-polymerase delta-interacting protein 2 (Poldip2) was significantly increased in the mouse cerebral cortex. The vascular permeability of Poldip2^+/+^ mice was significantly increased, while that of Poldip2^+/−^ mice was significantly reduced. In addition, the NF-κB/Cox2 signaling in the cerebral cortex of Poldip2^+/−^ mice is inhibited. Cox-2 is a key mediator of arachidonic acid metabolism and can promote the synthesis of PGE2 (Minghetti, 2004). PGE2 is also down-regulated in the cerebral cortex. However, opposite results were observed in Poldip2^+/+^ mice. We also observed that siPoldip2 significantly reduced LPS-induced endothelial cell permeability *in vitro*. This suggests that the Poldip2/COX-2/PGE2 signaling mediates changes in endothelial cell permeability in septic mice (Kikuchi et al., 2019). Inflammation also affects tight junctions in endothelial cells, which are maintained by important tight junction proteins, such as Claudin-5, Occludin, ZO-1, to preserve the integrity of the BBB (Sandoval and Witt, 2008). The study found that activation of NLRP3 after LPS treatment in the BBB *in vitro* model, which was constructed by co-culturing mouse CMECs and astrocytes, promoted inflammation, disrupted tight junctions, and reduced expression of tight junction-related proteins (Occludin, Claudin-5 and ZO-1) (Chen S. et al., 2020). In addition, the causes of nerve damage previously described, such as pyroptosis, mitochondrial dysfunction, and ferroptosis, also contribute to disruption of the BBB. We have summarized the pathogenesis of SAE in Figure 1 and Table 1.

**Table 1:** The pathological damage of SAE sepsis-associated encephalopathy.

	Ref.	Model	Test sites	Biochemical measurements
Neuroinflammation	Mei et al. (2021)	CLP	Hippocampus	TNF-α, IL-6, and IL-1β ↑ α2A-adrenoceptors (astrocytes) ↓
	Fang et al. (2022)	CLP	Cortex	NLRP3 and IL-1β ↑
	Shen et al. (2021)	CLP	Hippocampus	IL-1β, IL-6 ↑; microglial (M1 phenotype) ↑
	Saito et al. (2021)	CS	Cortex	IL-1β, IL-6 ↑; microglial, neutrophils, lymphocyte, CD 4^+^ T cell, CD 8^+^ T cell, Treg, Th2 ↑
	Ding et al. (2022)	CLP	Hippocampus	NLRP3, IL-1β (CMECs) ↑; TNF-α, IL-1R1, pNF-κB (microglial) ↑
	Wang et al. (2022b)	CLP, LPS	Hippocampus; microglial cells (BV-2)	IL-1β, TNF-α, p65 (microglial) ↑; Foxc1, IκBα (microglial, hippocampus) ↓
	Zou et al. (2022)	CLP	Cortex and hippocampus Primary microglia and astrocyte	miR-146a, TLR7 ↑;
Oxidative stress	Zhang H. et al. (2022)	CLP, LPS	Hippocampus; primary microglia	i-NOS, COX-2 and Nrf2/HO-1 (microglial) ↑
	Ding et al. (2022)	CLP	Hippocampus	i-NOS (microglial) ↑; ROS (CMECs) ↑
	Wang J. et al. (2022)	CLP	Hippocampus	ROS, MDA ↑; GSH ↓
	Wei et al. (2022)	CLP	Cortex; CMECs (bEnd.3)	ROS, MDA ↑; GSH ↓
	Haileselassie et al. (2020)	LPS	Brain CMECs, astrocyte and primary neuron	ROS, MitoSox, H_2_O_2_ ↑
	Xie et al. (2022)	CLP	Brain	MDA ↑
Autophagy	Zhuang et al. (2020)	CLP, LPS	Hippocampus microglial cells (BV-2)	TNF-α, IL-6, HMGB1 ↑; microglial (M1 phenotype) ↑; p-mTOR/mTOR, p62 (BV-2) ↑
	Shen et al. (2021)	CLP	Hippocampus	CXCR5 ↑
	Xie et al. (2022)	CLP	Hippocampus	LC3B, LaminB1 ↑
	Xi et al. (2021)	CLP	hippocampal neurons (H19-7)	The ratio of LC3II/I ↑
Neuronal damage	Xu et al. (2019)	CLP	Brain	Caspase-1, GSDMD, GSDMD-NT ↑; IL-1β, MCP-1, TNF-α ↑
	Fu et al. (2019)	CLP	Hippocampus	NLRP3, Caspase-1, GSDMD ↑; IL-1β, IL-18 ↑
	Chen S. et al. (2020)	LPS	Cortex; CMECs, astrocytes	Bax, GSDMD (*in vitro*) ↑; IL-1β, IL-18, NLRP3, p65 (*in vitro*) ↑
	Wang J. et al. (2022)	CLP	Hippocampus	GPX4, ACSL4, SLC7A11 ↑
	Wei et al. (2022)	CLP	Cortex; CMECs (bEnd.3)	Fe ion, Serum exosome-packaged NEAT1 ↑; TFRC, GOT1 ↑; miR-9-5p, GPX4 ↓
	Yin et al. (2020)	CLP	Hippocampus	SOX2OT, SOX2 ↑
	Qi et al. (2023)	CLP	Hippocampus	ptau, tau oligomer, TTBK1, ptau key kinase ↑
	Tang et al. (2023)	CLP	Hippocampus	Munc18-1, Syntaxin1A, Synaptophysin ↑
	Xie et al. (2022)	CLP	Brain	Transferrin, GPX4, GSDMD-NT ↑ pro-caspase-1, caspase-1 p20 ↑
	Xi et al. (2021)	CLP	Hippocampus	Bax, cleaved-caspase 3 ↑; Bcl-2 ↓
Organelle dysfunction	Wang J. et al. (2022)	LPS	Hippocampal neuronal cell line (HT-22)	MMP ↓
	Haileselassie et al. (2020)	LPS	Brain CMECs, astrocyte and primary neuron	p Drp1, p53, glycolysis ↑ MMP, ATP, oxidative phosphorylation ↓
	Ding et al. (2022)	CLP	CMECs	LC3-II, ROS ↑; p62, mitophagy ↓
	Li et al. (2020)	LPS	neuronal cell (PC12 and MES23.5)	UPR markers (GRP78, CHOP and PERK) ↑
Imbalance of neurotransmitters	Lee et al. (2023)	LPS	Hippocampus	Glutamate ↑
	Xie et al. (2022)	CLP	Brain	Glutamate, SXC, NR2B ↑
	Tang et al. (2023)	CLP	Hippocampus	Glutamate, NMDAR1 ↑
	Ge et al. (2023)	CLP	Prefrontal cortex	NMDAR, AMPAR, CaMKIIa、pCREB, BDNF ↑
	Zhu et al. (2016)	LPS	Hippocampus	AChE ↑; ChAT, CHRM1,ACh ↓
	Field et al. (2012)	LPS (mu-p75-sap)	Hippocampus	AchE, ChAT ↓
	Zhang et al. (2023)	LPS	Hippocampus	MMP-9 ↑; PNN, PV, gamma oscillations ↓
BBB disruption	Zou et al. (2022)	CLP	Brain	BBB permeability, TLR7 ↑
	Haileselassie et al. (2020)	LPS	Brain CMECs, astrocyte and primary neuron	BBB permeability, VCAM-1, ICAM-1 ↑ ZO-1, occludin ↓
	Kikuchi et al. (2019)	LPS	Cortex Primary CMECs	Poldip2, p65, Cox-2, PGE2 ↑
	Chen S. et al. (2020)	LPS	Cortex CMECs, astrocyte	Occludin、Claudin-5, ZO-1, Bcl-2 ↓ Maf1, p65, NLRP3, Bax, GSDMD ↑

↑, upregulated by intervention; ↓, downregulated by intervention. ACh, acetylcholine; AChE, acetylcholinesterase; BBB, Blood–brain-barrier; ChAT, choline acetyltransferase; CHRM1, muscarinic acetylcholine receptor-1; CLP, the cecal ligation and puncture; CMECs, cerebral microvascular endothelial cells; CS, cecal slurry; GSH, glutathione; LPS, lipopolysaccharide; MDA, malondialdehyde; MMP, mitochondrial membrane potential; mu-p75-sap, murine-p75-saporin; NR2B, N-methyl-D-asperate receptor subunit; PNN, perineuronal net; PV, parvalbumin; SXC, System xc-; UPR, unfolded protein response.

## 3 Interaction between brain and peripheral organs in sepsis

### 3.1 Brain and gut microbiota

The disorders of gut microbiota mediated multiple organ damage associated with sepsis (Cryan et al., 2019). Microbial metabolites are increasingly recognized as key mediators in the functional effects of gut-brain communication. Due to various reasons, such as the use of broad-spectrum antibiotics, patients with sepsis may experience severe disruption in their intestinal flora distribution, resulting in dysbiosis that can have a negative impact on prognosis (Wei et al., 2021). A retrospective study revealed that the 28-day mortality rate of patients with SAE was significantly higher compared to those non-SAE. Additionally, the incidence of gastrointestinal infection was found to be significantly higher in the SAE group compared to the non-AE group (Chen J. et al., 2020). A clinical study conducted 16S rDNA sequencing on fecal samples obtained from 20 patients with SAE. The study revealed that these patients exhibited a decrease in both the diversity and number of their gut microbiota (Wang et al., 2022a). Acupuncture and Chinese herbal medicine can improve related intestinal symptoms by regulating the type of intestinal flora and the level of neurotransmitters such as 5-hydroxytryptamine (5-HT) (Ling et al., 2022; Sun et al., 2023). However, the role of gut microbiota in modulating sepsis-associated encephalopathy (SAE) is still not well understood. Previous research has shown that fecal microbiota transplantation (FMT) is a promising method to treat gut dysbiosis and enhance brain function in septic rats (Li et al., 2021). The study found considerable individual variation in the neurological reflexes of septic mice 36 h after CLP. Mice with a neurological score > 6 had lower mortality and were defined as SAE-resistant mice (SER), while those with a neurological score ≤ 6 were defined as SAE-susceptible mice (SES). There was a notable decrease in the abundance and variety of the gut microbiota in SES mice, whereas the gut microbiota in SER mice exhibited slight dysregulation with notable differences in composition among individuals. Additional FMT experiments exposed that the gut microbiota from SER mice conferred a survival benefit following CLP, leading to marked reductions in IL-1β and TNF-α levels in both the serum and cerebral cortex of the mice. These findings provide evidence that the gut microbiota can transfer neuroinflammation induced by CLP (Fang et al., 2022). Another study found that administering the probiotic *Clostridium butyricum* (Cb) by gavage after CLP treatment significantly reduced cognitive disorders and neuronal damage. Additionally, the excessive activation of microglia and the elevation of BDNF levels were significantly suppressed by Cb. Moreover, Cb effectively ameliorated gut dysbiosis in SAE mice (Liu et al., 2020).

Although it has been established that the dysfunction of gut microbiota may be one of the important causes of SAE, and the brain-gut axis (signaling between the gut microbiota and the brain through the neuroendocrine immune network) plays an important role in this process (Mayer et al., 2015), the exact mechanism remains to be elucidated. The gut-microbiota-brain axis could be influenced significantly by the vagus nerve. Previous research reveals that LPS mice treated with FMT showed improved spatial memory. Moreover, there was a notable decrease in the levels of IL-1β, IL-6, and TNF-α within the cerebral cortex, along with a decrease in the count of Iba-1-positive microglia. Nevertheless, the advantageous outcomes achieved through FMT were nullified once cervical vagotomy was performed (Li et al., 2018). Another study found that exposure to red light worsened learning disabilities and anxiety-like behaviors in aged septic mice, while also altering the diversity and composition of the fecal microbiota (increasing Bacteroidetes abundance while decreasing Firmicutes abundance). The same behavioral deficits were observed in pseudo germ-free mice transplanted with fecal suspensions from septic mice exposed to red light. However, it is noteworthy that subdiaphragmatic vagotomy reversed these behaviors deficits (Xie B. et al., 2020). This suggests that dysbiosis of the gut microbiota following sepsis signals through the cervical and subdiaphragmatic vagus nerves, leading to cognitive impairment and anxiety-like behavior.

Other studies have speculated that the gut microbiota may regulate the local immune response in mesenteric lymph nodes (MLN) through intestinal epithelial cells (IEC), thereby influencing the progression of SAE. Intestinal epithelial cells (IECs) have been found to possess the capability of releasing extracellular vesicles, specifically exosomes, that have immunological activity. These exosomes are able to transport crucial molecules like proteins, DNA, and various inflammatory cytokines. Ultimately, they play a significant role in shaping the immune environment (Xu et al., 2016). *In vitro*, exosomes from IECs of SAE rats induced M1 macrophages polarization and increased IL-1β levels, while exosomes secreted by FMT-treated CLP rats showed the opposite results. Exosome secretion inhibitor (GW4869) significantly suppressed M1-type macrophage polarization and IL-1β expression in MLN. More importantly, it reduced serum and hippocampal IL-1β levels, and attenuated hippocampal damage, apoptosis and autophagy. The results are supported by the use of recombinant IL-1β and IL-1β antagonists. Furthermore, the supernatant of macrophages treated with IEC exosomes from SAE rats was co-cultured with neurons (H19-7), they found that microglia activation, apoptosis (Bcl-1, Bax, etc. protein), and autophagy (LC3II/I ratio) were present. However, IL-1β antagonists inhibited apoptosis and autophagy, which this was reactivated by rapamycin (RPA). These findings propose that the disruption of the gut microbiota might enhance the liberation of exosomes that originate from IEC, thereby having an impact on cognitive decline, inflammation, and damage to the hippocampus in SAE. This occurs through the polarization of M1 and the excretion of IL-1β in MLN, which leads to the impairment (Xi et al., 2021).

The metabolites of gut microbes into the bloodstream and subsequently into the brain, this is a pathway through which gut microbes establish a connection with the brain (Mayer et al., 2015). Several studies screened for differential metabolites in blood and brain of CLP mice, with l-gulono-1, 4-lactone levels decreasing in the blood and increasing in the brain (Han et al., 2023). Nonetheless, they possess the capability to transform into ascorbic acid (AA), which exhibits the potential to mitigate oxidative stress and endothelial dysfunction by augmenting endothelial NO synthesis. Therefore, their decrease in the blood is harmful (Kim et al., 2006), while the increase in the brain may be attributed to disruption of the BBB, leading to infiltration of macromolecules and entry into the brain. A prospective multicenter cohort study was conducted on 63 patients with sepsis to analyze their metabolic profile. The untargeted metabolomic analysis showed significant dysregulation of amino acid metabolism in sepsis patients. When compared to 43 normal controls, the sepsis multi-omics network exhibited key molecular changes associated with tryptophan biosynthesis (Chen et al., 2022). Tryptophan is crucial for maintaining immune homeostasis and optimizing gut barrier function. The metabolism of tryptophan, a precursor of 5-HT, can be modulated by the gut microbiota. Reduced levels of 5-HT in the brain can lead to abnormal behaviors such as anxiety, neurotic hallucinations, and insomnia (Roager and Licht, 2018). However, studies have found reduced levels of 5-HT in the blood and brain of CLP mice, which may be caused by disturbances in intestinal flora (Han et al., 2023). Another study focused on short-chain fatty acids (SCFAs), which are major fermentation metabolite of intestinal anaerobic bacteria. G protein-coupled receptor 43 (GPR43) interacts with SCFAs to exert anti-inflammatory effects in the central nervous system. The study revealed lower levels of acetate and propionate in the feces of SAE mice, as well as a decrease in the number of bacteria that produce SCFAs. After intragastric administration of SCFAsto SAE mice, it was found that SCFAs inhibited sepsis-induced cognitive disorders and neuroinflammation. However, the GPR43 antagonist (GLPG0974) counteracted the cognitive protective and anti-neuroinflammatory effects of SCFA (Liao et al., 2022).

### 3.2 Brain and liver metabolite

In addition to metabolites produced by gut microbes, the ketone body β-hydroxybutyrate (BHB), an intermediate metabolite in the liver during fat oxidation metabolism, contributes to preventing cognitive impairment after sepsis. Studies have found that BHB levels in the hippocampus were significantly reduced and blood BHB levels were increased after CLP treatment, but other studies found that blood BHB levels were decreased during sepsis (Lanza-Jacoby et al., 1990; Umbarawan et al., 2017). After subcutaneous injection of BHB, it was found that the levels of BHB in both blood and hippocampus were significantly increased, indicating that subcutaneous injection of BHB can penetrate the bloodstream and brain. The following study found that subcutaneous injection of BHB improved neuroplasticity and reduced mRNA levels of hippocampal IL-1β and TNF-α, as well as the percentage of microglial activation in the CA1 region and dentate gyrus in mice that survived CLP. In addition, a significant decrease in peripheral blood leukocyte count and neutrophil percentage. Intracerebroventricular injection of BHB also resulted in decreased mRNA levels of hippocampal IL-1β and TNF-α. *In vitro*, it was further found that both HCA2 (BHB receptor) and MCT2 (BHB transporter) played a role in improving the BHB response to LPS-induced neuronal injury and inflammation. Additionally, the inflammatory response was more significantly impacted by HCA2 than by MCT2. Since BHB is considered as an alternative energy source for the brain during states of energy deficiency, the study also examined the ADP/ATP ratio in the hippocampus during sepsis. However, there was no significant change observed after administering BHB. These findings propose that the administration of BHB leads to a decrease in both neuroinflammation and peripheral inflammation in mice subjected to CLP. Importantly, it is suggested that this reduction occurs via the activation of HCA2 and MCT2 pathways (Wang et al., 2020). This also indicates that the immune system disorder in sepsis affects the level of liver metabolites in circulation, which further impacts the occurrence of neuroinflammation in SAE.

## 4 Interaction between brain and peripheral neuroimmune system in sepsis

### 4.1 Cholinergic pathways

We previously introduced the role of the vagus nerve in the brain-gut axis, and here we continue to discuss the regulatory mechanism of the vagus nerve and its associated neurotransmitters in sepsis. The vagus nerve, which is the longest and most widely distributed of the 12 pairs of cranial nerves, is also the most important parasympathetic nerve. Furthermore, apart from its crucial involvement in controlling visceral function, the vagus nerve also possesses anti-inflammatory capacities, recognized as the cholinergic anti-inflammatory pathway (Borovikova et al., 2000). The involvement of cholinergic anti-inflammatory pathways in both CNS and peripheral inflammatory diseases has been extensively documented (Lee et al., 2010; Kolgazi et al., 2013). The CNS interacts bidirectionally with the immune system through the vagus nerve. The vagus nerve converts peripheral inflammation into nerve signals, which then enters the brain through afferent nerves. The brain immediately senses peripheral inflammatory stimuli and regulates peripheral immune responses through cholinergic efferent nerve fibers. Therefore, the vagus nerve acts as a “bridge” connecting peripheral inflammatory stimuli to the central nervous system (Olofsson et al., 2012).

A prospective, single-center study was conducted to enroll 45 patients with sepsis. The study observed that around one-third of sepsis patients with suspected SAE experienced a time-dependent increase in AChE activity in their blood samples. This increase in acetylcholinesterase activity results in the breakdown of acetylcholine, leading to cholinergic defects (Zujalovic et al., 2020). A research has investigated elements of cholinergic nerve activity in the hippocampus, such as acetylcholine transferase (ChAT), receptor-1 for muscarinic acetylcholine (CHRM1), acetylcholinesterase (AChE), and acetylcholine, in septic rats experiencing neuroinflammation in the hippocampus. It was found that LPS treatment resulted in the inhibition of ChAT and CHRM1 mRNA and protein expression in the hippocampal region of rats, decreased ACh concentration (measured by LC–MS/MS), enhanced AChE activity, increased TNF-α and IL-1β mRNA and protein expression, neuronal apoptosis (measured by TUNEL) and cognitive impairment. These results were reversed after administration of the acetylcholinesterase inhibitor huperzine (HupA). The effects of HupA include inhibiting the hydrolysis of AChE and promoting the expression of ChAT and CHRM1. It is suggested that the occurrence of inflammation in sepsis leads to the damage and dysfunction of cholinergic neurons, while HupA treatment improves SAE by promoting cholinergic nerve function and anti-inflammatory ability (Zhu et al., 2016). In another study examining electrophysiological changes in the hippocampus during sepsis, hippocampal function and long-term potentiation of excitatory synapses (LTP) in rat brain slices were studied using whole-cell patch-clamp single-cell electrophysiology techniques. The data showed that disruption of synaptic plasticity in the rat brain after LPS treatment was accompanied by an increase in after-hyperpolarization (AHP) mediated through small-conductance Ca^2+^-activated potassium channels (SK). Inhibiting SK channels can partially restore sepsis-induced deficits in synaptic plasticity. This can be done by using an SK2 channel blocker like apamin, a highly selective muscarinic M1 receptor allosteric agonist such as TBPB, or by increasing the lifespan of endogenous acetylcholine through a cholinesterase inhibitor like physostigmine (Zivkovic et al., 2015). All of these studies have confirmed that sepsis impairs cholinergic nerve function in the hippocampus. Conceivably, damage to hippocampal cholinergic neurons could further affect the neural circuitry of the inflammatory reflex, impeding normal immunomodulatory function of peripheral immune cells that contain cholinergic receptors such as macrophages, neutrophils. Thereby exacerbating peripheral inflammation and causing multi-organ damage, thus creating a vicious cycle. However, further research is needed to understand the regulatory mechanisms.

In addition, numerous animal experiments have confirmed that the intervention of cholinergic anti-inflammatory pathways through peripheral injection of α7 nicotinic acetylcholine receptor agonists can improve heart, lung, and multiple organ dysfunction (Sallam et al., 2018; Shao et al., 2019; Wedn et al., 2019). The functioning of the heart significantly affects the perfusion of blood in the brain. Mean arterial pressure (MAP) is a crucial clinical factor that can predict the development of brain injury related to sepsis. While the brain has the ability to regulate blood flow on its own, if the mean arterial pressure falls below a certain threshold, it can lead to decreased perfusion of organs (Ge et al., 2022). According to a cohort study, the incidence of SAEs was found to increase when systolic blood pressure was less than 90 mmHg, diastolic blood pressure was less than 46 mmHg, mean arterial pressure was less than 65 mmHg, and lactate level was greater than 3.5 mmol/L (Zhao et al., 2022). According to the latest guidelines from the Surviving Sepsis Campaign, it is recommended to initiate fluid resuscitation and vasopressors as early as possible in order to achieve a MAP greater than 65 mm Hg and maintain organ perfusion (Singer et al., 2016). A prospective observational study was conducted to investigate the use of near-infrared spectroscopy (NIRS)-derived cerebral blood oxygenation index (COx) for monitoring autoregulation and determining optimal blood pressure in six selected SAE patients who were not sedated with pharmacological agents. The results indicate a strong correlation between brain autoregulation and neural status. It was observed that patients with lower GCS (Godzilla Coma Scale) had consistently higher hourly COx measurements, while patients with higher GCS had lower hourly COx measurements. This suggests that autoregulatory dysfunction may contribute to the pathophysiology of SAE (Rosenblatt et al., 2020). A prospective observational study conducted transcranial Doppler examination on 40 patients with sepsis. A positive correlation between pulsatile index (PI) and changes in peripheral vascular resistance. The results showed an increase in PI in delirium patients and a decrease in cerebral blood flow index (CBFi), suggesting that patients may have developed cerebral microcirculation disorders (Pierrakos et al., 2014). Therefore, impaired automatic regulation/cerebral perfusion is one of the more important pathological injuries in SAE patients.

### 4.2 Peripheral immune cells

Sepsis is triggered by infection and the disease develops rapidly, with an early feature being an uncontrolled inflammatory response (Singer et al., 2016). The activation of inflammatory signaling pathways, such as Toll-like receptors and NF-κB, stimulates immune cells to over-activate and release more inflammatory factors. This leads to cytokine cascade reactions and “cytokine storms,” which amplify systemic inflammatory responses and damage systemic organs (Chousterman et al., 2017). Therefore, immune cells and their secreted cytokines play an irreplaceable role in the pathological process of sepsis.

A retrospective study was conducted on 86 patients diagnosed with severe sepsis. The study revealed that the percentage of cluster of differentiation CD4^+^ T lymphocyte clusters in the blood samples of patients with severe SAE was lower, and CD4^+^/cluster of differentiation CD8^+^ ratio was also decreased. Additionally, the study found a higher percentage of NK cells in these patients. This study provide evidence that immune imbalance plays a crucial role in the development of SAE (Lu et al., 2016). Following neuroinflammatory damage to the brain, peripheral secondary lymphoid organs have the potential to discharge abundant quantities of lymphocytes, encompassing T cells and B cells (Lewis et al., 2019). These immune cells are recruited from circulation to the brain along with many other peripheral immune cells. Brain-infiltrating T cells contribute to the recovery of depressive symptoms by addressing neuroinflammation (Ito et al., 2019). Nonetheless, limited knowledge persists regarding the mechanisms underlying T cell infiltration into the brain and their contribution to SAE progression. Investigating the impact of Cecal Serous (CS) treatment, the research revealed a substantial rise in both CD4^+^ and CD8^+^ T cell populations within the cerebral cortex of mice. In addition, the accumulation of T cells in the brains of CS mice was predominantly composed of naïve T cells, rather than effector memory T cells. Conversely, there was a noteworthy decrease in T cells in the peripheral blood and spleen of CS mice, which is a characteristic manifestation of sepsis-induced immunosuppression (Hotchkiss and Karl, 2003; Saito et al., 2021). They subsequently discovered that an increase in Tregs was only observed in cervical lymph nodes (CLN) among the numerous secondary lymphoid tissues in CS mice, suggesting that CLN may serve as an important source for T cell recovery following severe suppression caused by sepsis. FTY720, an antagonist of sphingosine-1-phosphate, inhibits the drainage of lymphocytes from lymph nodes, thereby preventing T cells from migrating into the brain. After intraperitoneal injection of FTY720, FTY720 administration was found to decrease the number of CD4+ and CD8+ T cells in the brains of CS mice. Moreover, a notable decline was observed in the concentrations of anti-inflammatory cytokines IL-4 and IL-10 expounded by T cells within the cerebral cortex. These changes lead to increased expression levels of IL-1β and TNF-α in the cerebral cortex and blood of CS mice, as well as an increase in microglia and a decrease in astrocytes in the cortex. This suggests that FTY720 delays the recovery of anxiety-like behavior and the expression of persistent neuroinflammation. Therefore, T cells derived from CLN enter the brain from circulation and contribute to attenuating neuroinflammation and recovering anxiety-like behavior (Saito et al., 2021).

The precursor of brain-derived neurotrophic factor (pro BDNF) is a distinct protein compared to mature BDNF. Signaling via pro BDNF-p75NTR promotes the apoptosis of neurons and axon pruning, while concurrently exerting negative control over learning and memory (Teng et al., 2005). Immunofluorescence detection revealed that proBDNF was upregulated in meningeal and peripheral blood immune cells (CD3^+^ T cells, CD4^+^ T cells, CD19^+^ B cells) after LPS injection. Additionally, a significant decrease in the proportion of CD4^+^ T cells was observed in splenocytes collected from septic mice that were treated with exogenous proBDNF protein. The subsequent intraperitoneal injection of proBDNF antagonist (McAb-proB) significantly restored the percentage of CD4^+^ T cells in the meninges to normal levels and reversed the downregulation of anti-inflammatory cytokines IL-4, IFN-γ and IL-13 mRNA as well as the upregulation of pro-inflammatory cytokines IL-1β and IL-6 mRNA in the meninges. However, it is worth noting that the intracerebroventricular injection of McAb-proB did not play any role. The evidence presented indicates that the immune system’s upregulation of proBDNF plays a role in the development of SAE. This occurs through the downregulation of circulating CD4+ T cells, thereby restricting their infiltration into the meninges. Additionally, it disrupts the balance between pro-inflammatory and anti-inflammatory factors within the meninges, leading to a disturbance in their homeostasis (Luo et al., 2020).

The SAE may also be affected by cytokines released from peripheral immune cells. The study found that the upregulation of IL-17R was accompanied by microglia activation (increased Iba-1 fluorescence intensity) in the brain tissue of CLP mice, as observed through an immunofluorescence assay. After administering of recombinant IL-17A through intraventricular injection, there was observed an elevation in the levels of pro-inflammatory cytokines (specifically, IL-1β and TNF-α) within the brain region. This led to a marked increase in the activation of microglia specifically within the hippocampus. Conversely, when both anti-IL-17A and anti-IL-17R antibodies were administered through intraventricular injection, there was a notable reduction in central nervous system (CNS) inflammation and a subsequent inhibition of microglial activation. These findings were further corroborated by *in vitro* experiments, thereby highlighting the influence of the IL-17A/IL-17R signaling pathway on microglial activation (Ye et al., 2019). Notably, earlier studies by the team have shown that IL-17A secreted by peritoneal γδ T cells rapidly enters the circulation in the early stages of sepsis. Intraperitoneal blockade of IL-17A reduces pro-inflammatory cytokines and neutrophil infiltration in alveolar lavage fluid, thereby ameliorating lung injury and improving survival (Li et al., 2012). Their two studies suggest that activation of peritoneal γδ T cells leads to the secretion of IL-17A, causing lung injury. In the presence of BBB injury, circulating IL-17A may also be transferred to the CNS and exacerbate SAE. However, further studies are still needed to establish it. We speculate that cytokines released by peripheral immune cells, such as macrophages and neutrophils, in addition to peritoneal γδ T cells, may worsen SAE by damaging the BBB.

**Figure 2:**
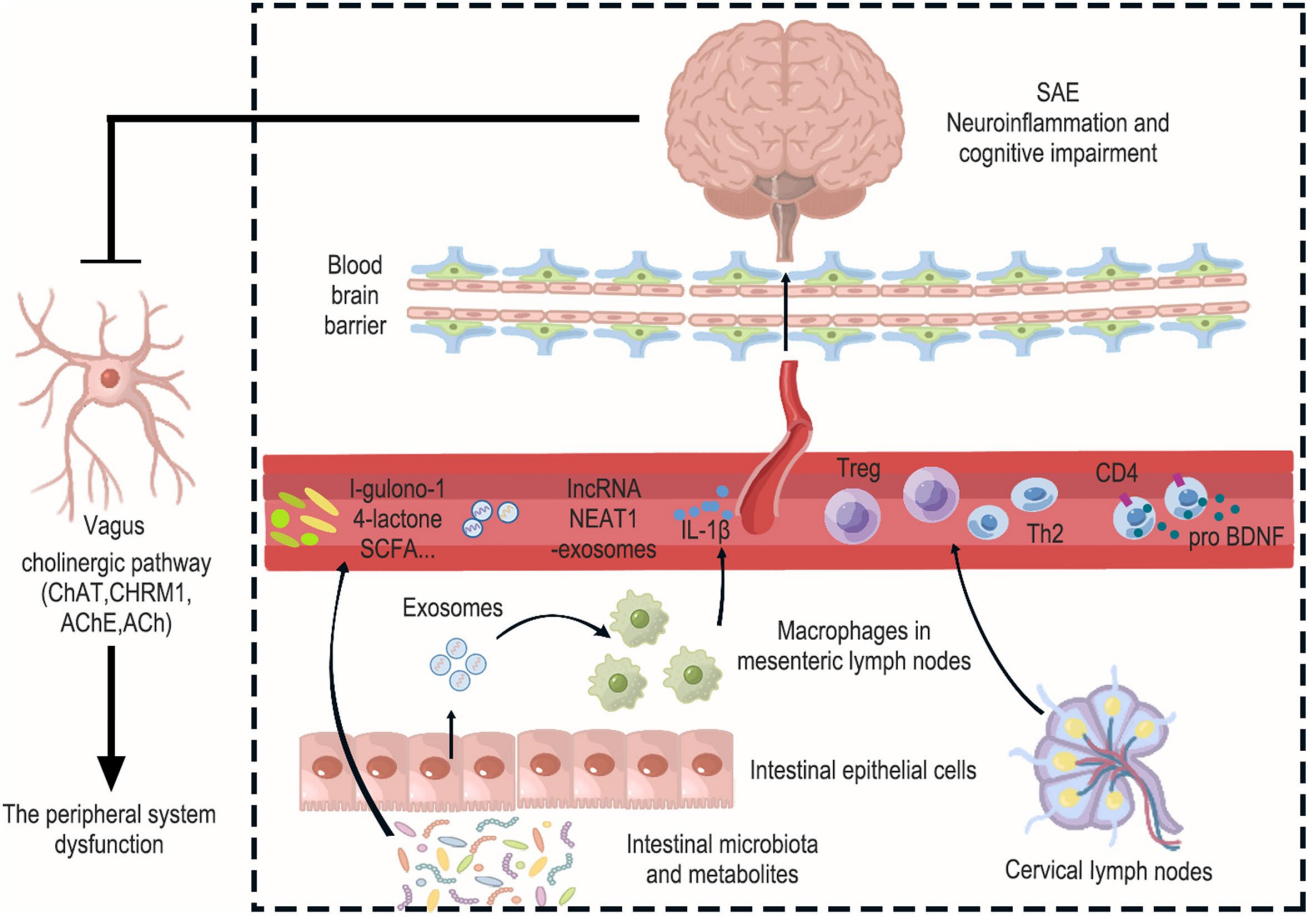
Interactions between the brain and peripheral immunity in sepsis. Peripheral system dysfunction is a crucial factor that should not be overlooked in the context of SAE. This encompasses disorders related to intestinal flora and their metabolism, the release of pathogenic exosomes from intestinal epithelial cells, the release of inflammatory factors from mesenteric immune cells, and alterations in the function of peripheral immune organs like cervical lymph nodes. Specifically, Levels of the gut microbiota metabolite l-gulonone-1,4-lactone were found to decrease in the blood and increase in the brain. Additionally, the number of bacteria producing short-chain fatty acids (SCFAs) in the gut microbiota decreased. This is significant because SCFAs have the ability to inhibit neuroinflammation. Furthermore, the expression of lncRNA NEAT1 increased in exosomes and the cerebral cortex, suggesting that exosomes carrying lncRNA NEAT1 may cross the blood–brain barrier and reach the cerebral cortex. And intestinal epithelial cell-derived exosomes promote M1 macrophage polarization and IL-1β production in mesenteric lymph nodes, leading to damage to hippocampal neurons. Interestingly, Treg cells and Th2 cells were found to increase in cervical lymph nodes (CLN), and these cells also showed an increase in the cerebral cortex. This suggests that T cells from the CLN may enter the brain from the circulation and have the ability to suppress neuroinflammation. Moreover, the upregulation of proBDNF in the immune system was found to suppress the number of circulating CD4^+^ T cells and limit their infiltration into the meninges. Finally, the occurrence of neuroinflammation in the brain was observed to inhibit cholinergic anti-inflammatory pathways, which was evident through increased AChE and decreased ChAT, CHRM1, and ACh in the hippocampus. Collectively, these changes exacerbate peripheral system dysfunction.

In some studies, we have found that exosomes (Exo) are the most potential carriers for peripheral immune cells to act on the brain through the blood. Mesenchymal stem cells (MSC) have become clinically valuable therapeutic tools due to their high potential for differentiation, capacity for proliferative, and ability to modulate the immune system (Shetty et al., 2010). It was found that the injection of umbilical cord MSCs (UC-MSCs) via femoral vein significantly reduced the expression levels of TNF-α, IL-6 and HMGB1 as well as microglia activation (expression of Iba-1 markers) in brain tissue of LPS-treated mice. This improved cortical neuronal damage and cognitive impairment (Zhang Z. et al., 2022). Some studies suggested that MSCs can migrate into the brain through paracellular or transcellular pathways (Schmidt et al., 2006). It is possible that activation of endothelial cells and astrocytes in SAEs leads to decreased tight junctional integrity and reduced formation of the paracellular space, allowing cells to migrate via paracellular pathways (Liu et al., 2013). Nonetheless, the blood–brain barrier continues to be a significant physical barrier that stem cells need to overcome (Ballabh et al., 2004). In comparison, exosomes at the nanoscale (30-150 nm) can more easily penetrate the BBB while still exhibiting the same immunomodulatory and regenerative capabilities as their parent cells (Timmers et al., 2007). The role of exosomes is to enhance the communication between cells by transferring various components including cell surface receptors, cytokines, lipids, and RNA molecules from the donor cells to the recipient cells (van Niel et al., 2018). It has been demonstrated that intranasally administered MSC-Exo reach the brain and reduce microglia-mediated neuroinflammation in rats with perinatal brain injury (Thomi et al., 2019). Another piece of evidence suggests that exosomes can participate in the pathological process of SAE through the BBB. In this study, exosomes were purified from CLP-treated rat cerebral cortex and exosome markers including TSG101, CD9, and CD63 were identified. To further investigate whether exosome packaging is the primary mode of delivery of lncRNA NEAT1, the expression of lncRNA NEAT1 in exosomes and cerebral cortex was examined by the researchers. Using qRT-PCR, it was found that the expression of lncRNA NEAT1 remained consistent in both exosomes and cerebral cortex. Moreover, an observable increase in lncRNA NEAT1 expression was observed in rats treated with CLP, and there was a positive correlation between their lncRNA NEAT1 expression levels in the cerebral cortex and exosomes. This suggests that exosomes carrying lncRNA NEAT1 can cross the blood–brain barrier and reach the cerebral cortex, promoting ferroptosis. In addition, it remains to be explored whether are the primary secretion of peripheral immune cells, serving as the main means of transporting lncRNA NEAT1 to the cerebral cortex (Wei et al., 2022). In conclusion, peripheral immune cells may be more involved in the pathological process of SAE through their derived exosomes. Finally, we summarized the interaction between the brain and peripheral system in sepsis in Figure 2 and Table 2.

**Table 2:** Interaction between peripheral system dysfunction and sepsis-associated encephalopathy.

	Ref.	Model	Test sites	Biochemical measurements
Gut microbiota	Fang et al. (2022)	CLP (FMT)	Cortex Serum	IL-1β, TNF-α, NLRP3 ↓; IPA ↑
	Liu et al. (2020)	CLP	Brain	Iba-1 ↑; BDNF ↓; gut microbiota dysbiosis
	Li et al. (2018)	LPS (FMT)	Cortex Hippocampus	Iba-1 (Cortex) ↓; TNF-α, IL-6, IL-1β (Hippocampus) ↓
	Xie B. et al. (2020)	LPS	Fecal microbial	Chao 1 index, Shannon index, ACE index ↓
	Xi et al. (2021)	CLP (FMT)	Hippocampus	IL-1β, IL-6, TNF-α, IBA-1 ↓; M1-type macrophage (MLN) ↓;
	Han et al. (2023)	CLP	Brain Fecal microbial	TNF-α, IL-6, HMGB1 ↑; Tryptophan ↓; Changes in the composition of gut microbiota
	Liao et al. (2022)	CLP	Hippocampus Fecal microbial	IL-1β, IL-6, TNF-α ↓; Acetate, propionate ↓; Relative abundance of SCFAs-producing bacteria ↓
Liver metabolite	Wang et al. (2020)	CLP	Hippocampus	BHB, neuroplasticity ↓; IL-1β, TNF-α, IBA-1 ↑;
Cholinergic pathways	Zhu et al. (2016)	LPS	Hippocampus	AChE ↑; ChAT, CHRM1,ACh ↓
	Zivkovic et al. (2015)	LPS	Hippocampus	AHP, SK channels ↑; LTP ↓
Peripheral immune cells	Saito et al. (2021)	CS	Cortex PB Spleen CLN	Neutrophils, lymphocyte, CD 4^+^ T cell, CD 8^+^ T cell, Treg, Th2 (Cortex) ↑; T cells (Spleen), CD 4^+^ T cell, CD 8^+^ T cell (PB) ↓ Treg, Th2 (CLN) ↑;
	Luo et al. (2020)	LPS	Meninges PB	IL-1β, IL-6 (meninges) ↑; IL-4, IFN-γ, IL-13 (meninges) ↓; CD3^+^ T cell, CD4^−^CD8^+^ T cell, CD11b^+^ monocytes/macrophages (meninges) ↑; CD4^+^CD8^−^ Th cells, CD19^+^ B cells (PB) ↓; proBDNF (meninges, PB) ↑
	Ye et al. (2019)	CLP	Brain Hippocampus	IL-17A, IL-1β, TNF-α, IL-17R↑; CD11b, Iba-1 ↑
	Wei et al. (2022)	CLP	Cortex	lncRNA NEAT1, Exo- lncRNA NEAT1 ↑

↑, upregulated by intervention; ↓, downregulated by intervention. ACh, acetylcholine; AchE, acetylcholinesterase; AHP, after-hyperpolarization; BHB, the ketone body β-hydroxybutyrate; ChAT, choline acetyltransferase; CHRM1, muscarinic acetylcholine receptor-1; CLN, cervical lymph nodes; CLP, cecal ligation and puncture; FMT, fecal microbiota transplantation; HRV, heart rate variability; IPA, Indole-3-propionic acid; LPS, lipopolysaccharide; LTP, long-term potentiation; MLN, mesenteric lymph nodes; PB, peripheral blood; SK, small-conductance Ca^2+^-activated potassium.

## 5 Summary

In summary, the pathogenesis of SAE is broadly consistent with previous studies, mainly including microglia activation, programmed cell death (apoptosis, pyroptosis and ferroptosis), neuronal loss, mitochondrial damage and functional barrier, endoplasmic reticulum stress, neurotransmitters imbalance (glutamate, acetylcholine and γ-aminobutyric acid), as well as blood–brain barrier impairment due to altered endothelial permeability and disruption of tight junctions. With increasing attention to the multi-organ dysfunction caused by sepsis, there has been progressive demonstration of damage to organs such as the brain, intestinal tract, heart, and lungs. However, sepsis is a systemic inflammatory disease, and the interaction between organs during inflammation has received little attention. Therefore, we focus on the interplay between brain and peripheral system in sepsis. We discovered that several factors often exacerbate the condition of septic encephalopathy, including vagus nerve-mediated gut microbiota and its metabolites, exosomes secreted by intestinal epithelial cells, liver metabolites, cholinergic pathways, T cells and their cytokines in peripheral secondary lymphoid organs. Therefore, we suggest paying attention to the prevention and treatment of brain injury at all stages of sepsis to prevent a vicious cycle of uncontrolled systemic inflammation following CNS injury.

## Author contributions

JZ: Writing – original draft. SC: Writing – original draft. XH: Writing – original draft. LH: Writing – review & editing. PL: Writing – review & editing. XY: Visualization, Writing – review & editing. ZL: Writing – review & editing. JL: Writing – review & editing. LG: Writing – review & editing. ZC: Supervision, Writing – review & editing. YG: Supervision, Writing – review & editing. BC: Supervision, Writing – review & editing.

## Glossary

5-HT: 5-hydroxytryptamine
AA: ascorbic acid
AchE: acetylcholinesterase
AHP: after-hyperpolarization
AhR: aryl hydrocarbon receptor
BBB: blood–brain barrier
BHB: ketone body β-hydroxybutyrate
Cb: *Clostridium butyricum*
ChAT: acetylcholine transferase
CHRM1: muscarinic acetylcholine receptor-1
CLN: cervical lymph nodes
CLP: cecal ligation-punctured
CMECs: cerebral microvascular endothelial cells
CNS: central nervous system
COX-2: Cyclooxygenase-2
CS: Cecal Serous
ER: Excessive endoplasmic reticulum
FMT: fecal microbiota transplantation
GOT1: glutamate-oxaloacetate transaminase 1
GSH: glutathione
GPR43: G protein-coupled receptor 43
HupA: huperzine
IEC: intestinal epithelial cells
i-NOS: inducible nitric oxide synthase
IPA: indole-3-propionic acid
LPS: lipopolysaccharide
LTP: long-term potentiation
miRNA: microRNA
MDA: malondialdehyde
MLN: lymph nodes
MMP: mitochondrial membrane potential
NMDAR: Ionotropic glutamate receptors
PGE2: prostaglandin E2
pro BDNF: Brain-derived neurotrophic factor precursor
SAE: sepsis-associated encephalopathy
SER: SAE-resistant mice
SES: SAE-susceptible mice
SCFAs: short-chain fatty acids
SK: small-conductance Ca^2+^-activated potassium channels
TFRC: transferrin receptor
Treg: regulatory T cells
UC-MSCs: umbilical cord MSCs
UPR: unfolded protein response
